# Epigenomic Approaches for the Diagnosis of Rare Diseases

**DOI:** 10.3390/epigenomes6030021

**Published:** 2022-07-27

**Authors:** Beatriz Martinez-Delgado, Maria J. Barrero

**Affiliations:** 1 Molecular Genetics Unit, Institute of Rare Diseases Research (IIER), Spanish National Institute of Health Carlos III (ISCIII), 28220 Madrid, Spain; bmartinezd@isciii.es; 2 Centro de Investigación Biomédica en Red de Enfermedades Raras, CIBERER U758, 28029 Madrid, Spain; 3 Models and Mechanisms Unit, Institute of Rare Diseases Research (IIER), Spanish National Institute of Health Carlos III (ISCIII), 28220 Madrid, Spain

**Keywords:** molecular diagnosis, rare diseases, epigenetics, transcriptomics

## Abstract

Rare diseases affect more than 300 million people worldwide. Diagnosing rare diseases is a major challenge as they have different causes and etiologies. Careful assessment of clinical symptoms often leads to the testing of the most common genetic alterations that could explain the disease. Patients with negative results for these tests frequently undergo whole exome or genome sequencing, leading to the identification of the molecular cause of the disease in 50% of patients at best. Therefore, a significant proportion of patients remain undiagnosed after sequencing their genome. Recently, approaches based on functional aspects of the genome, including transcriptomics and epigenomics, are beginning to emerge. Here, we will review these approaches, including studies that have successfully provided diagnoses for complex undiagnosed cases.

## 1. Introduction

Epigenetics plays an important role in pathogenicity since it regulates basic cellular functions, such as gene expression, DNA damage, chromatin topology, and chromosomal organization. DNA in the eukaryotic cell nucleus is wrapped around two copies of each of the core histones (H2A, H2B, H3, and H4) to form chromatin. Among other epigenetic mechanisms, modifications of DNA and histones play critical roles in gene expression regulation. The level of chromatin compaction has important consequences for gene transcription as it influences the accessibility of DNA sequences to transcription factors and other regulatory proteins. Modifications of DNA and histones regulate the level of chromatin compaction, either directly or by facilitating the binding of remodeling proteins that recognize modified sites.

Genetic alterations can have an important impact on epigenetic regulation. Mutations might affect the function of genes involved in histone or DNA modifications or even affect histone genes. These alterations typically have a broad impact on gene expression. Alternatively, mutations can be located in regulatory elements or alter the conformation of chromatin affecting the expression of particular genes.

A disease is considered rare if it affects fewer than 1 in 2000 people [1]. Despite the low individual incidence, rare diseases affect altogether 350 million people in the world [2]. More than 8000 rare diseases have been described [3]. The large variabilities and complexities of symptoms often complicate their diagnoses, which can take up to several years for some patients [4]. Many rare diseases are associated with epigenetic alterations that cause changes in gene expression and can be used to aid diagnosis [5].

## 2. Epigenetic Aspects of Rare Diseases

**Figure 1:**
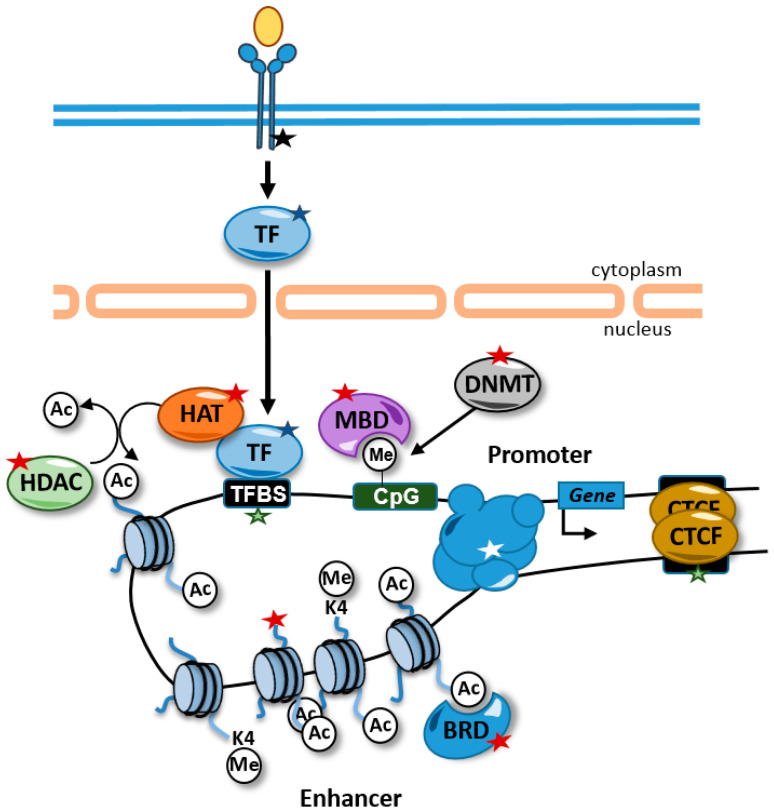
Alterations causing rare diseases that disrupt the epigenome and affect gene expression. Alterations in signal transduction pathways that regulate transcription factor activity (black star), transcription factors (blue star), transcription factor binding sites (green stars), chromatin-related activities (red stars), and promoter–enhancer interactions (white star) can affect gene expression. Some alterations, such as mutations in transcription factor binding sites, are likely to affect the expression of one gene, but other alterations, such as alterations in transcription factors and histone modifying enzymes, are predicted to have genome-wide impacts on the epigenome and in the expression of genes. For example, disruptions of transcription factor activity might interfere with the recruitment of HATs to the chromatin and maintain the proper levels of histone acetylation at enhancers. TFBS, transcription factor binding site; HDAC, histone deacetylases; HAT, histone acetyltransferases; BRD, bromodomain-containing protein; MBD, methyl CpG binding protein; DNMT, DNA methyltransferase; TF, transcription factor; Ac, acetylated residue; Me, methylated cytosine.

Alterations in chromatin properties and structure are common in rare diseases and can be used as diagnostic tools. These alterations can be caused directly by mutations in genes that encode proteins involved in the regulation of chromatin. In addition, other alterations not involving epigenetic factors directly can affect the epigenome. For example, chromatin-related factors are very often recruited to chromatin through transcription factors and, therefore, mutations in transcription factors, their binding sites, or components of signal transduction pathways that control their activity can also lead to alterations in the cellular epigenetic landscape (Figure 1).

Haploinsufficiency in chromatin-related factors frequently causes neurodevelopmental syndromes. Although most of these proteins are ubiquitously expressed, the nervous system appears to be particularly vulnerable to the alteration of their activities. Next, we review critical aspects of epigenetic regulation and its alterations (Table 1).

### 2.1. DNA Methylation

DNA methylation is catalyzed by DNA methyltransferases (DNMTs), typically at cytosines (5mC) [6]. Despite being a relatively stable mark, it can be reversed by the action of ten-eleven translocation (TET) enzymes that oxidize the methyl group of 5mC to yield 5-hydroxymethylcytosine (5hmC) [7]. DNA methylation is essential for normal development and is associated with a number of key processes, including genomic imprinting, X-chromosome inactivation, and gene repression. In particular, methylation of CpG islands, 500–2000 bp CpG-rich areas typically found near the transcription start site of genes, is an important mechanism for gene silencing [6]. The 5hmC residues are found in active genes and are emerging as regulators of gene activation and cellular differentiation during embryonic development and brain maturation [8].

The DNA-methyltransferase enzymes (DNMT1, DNMT3A, and DNMT3B) maintain normal patterns of DNA methylation. In addition, 5mC and 5hmC can be recognized by methyl binding proteins (MECP2, MBD1, MBD2, MBD3, MBD4, MBD5, and MBD6) that possess a methyl-binding domain (MBD) and act as methylation-sensitive transcriptional repressors. Both mutations in DNMTs and methyl binding proteins can cause rare syndromes (Table 1). Mutations in *DNMT1* are associated with neuropathies, mutations in *DNMT3A* cause overgrowth syndromes with intellectual disability, and *DNMT3B* mutations are involved in immunodeficiency and intellectual disability [9]. Loss-of-function mutations in *MECP2* cause Rett syndrome, a rare neurodevelopmental disorder, and alterations in other MBD-containing proteins have been described in autism spectrum disorders [10]. Since all these factors are involved in gene repression, it is expected that their loss-of-function results in the overexpression of certain genes that likely contribute to the disease. However, how the induction of genes contributes to the phenotype is not completely understood. In addition, other chromatin functionalities might be compromised. For example, mutations in *DNMT3B* cause centromeric instability and increased frequency of somatic recombination [11].

Mutations in factors controlling DNA methylation can also be involved in imprinting disorders. In humans, around 100 autosomal genes are preferentially expressed from only one of the two parental chromosomes as a result of differential DNA methylation during gametogenesis in the male and female germ lines [12]. Alterations in the methylation status of these genes, most commonly loss but also acquirement of DNA methylation at the non-imprinted locus, might be driven by genetic changes in a cis-acting element or trans-acting factor involved in the establishment or maintenance of imprinted methylation [13]. A number of alterations may also be caused by random environment-driven errors [13]. Most individuals with imprinting disorders exhibit altered DNA methylation at several imprinted loci, a condition that is referred to as multilocus imprinting disturbance (MLID). The molecular basis of these disorders is complex with few pathological variants likely involved in the establishment and maintenance of imprinting identified [14]. Genetic alterations that affect cis-acting elements might include deletions, duplications, and translocations, but perhaps are more common cases of uniparental disomy in which two copies of a given imprinted region are from one progenitor. Due to the dynamic regulation of DNA methylation in cells, it is relatively common for patients to show mosaicisms with variable levels of DNA methylation at imprinted regions between or within tissues, which might complicate the diagnosis. Emerging new technologies now allow the detection of allele-specific expression in single cells and are contributing to improving our understanding of how DNA methylation and epigenetics in general contribute to mosaicisms in rare diseases [15].

### 2.2. Histone Modifications

Dysregulation of histone methylation and acetylation have been involved in rare diseases [16]. Histone lysine methylation plays an essential role in gene expression and its deregulation has been linked to different neurodevelopmental conditions. Lysine methylation is a complex modification that affects gene expression in different ways depending on the modified residue [17]. Lysine methylation occurring at residues 4 and 36 of histone H3 is generally associated with active chromatin. Tri-methylation of histone H3 at lysine 4 (H3K4me3) is usually located at the transcription start sites (TSS) of actively transcribed genes while tri-methylation of histone H3 at lysine 36 (H3K36me3) is usually found at the gene bodies. Tri-methylation at lysine 9 and 27 of histone H3 (H3K9me3 and H3K27me3), and lysine 20 of histone H4 (H4K20me3) are typically associated with inactive or repressed chromatin. H3K27me3 is mediated by the polycomb repressive complex and is generally associated with facultative heterochromatin, while H3K9me3 marks constitutive heterochromatin. The levels of histone lysine methylation at a particular genomic location are dynamically controlled by the actions of histone lysine methyltransferases (KMTs) and demethylases (KDMs). Haploinsufficiency of KMTs or KDMs manifests in numerous neurodevelopmental disorders (Table 2) [18]. The overlap of symptoms caused by mutations in diverse histone modifiers and distinct symptoms caused by genes belonging to the same family of proteins suggests the existence of a complex network of gene expression regulation in the brain. The Kabuki syndrome can be caused by the loss of function of *KMT2D* (also called *MLL2*) or *KDM6A* (also called *UTX*). This overlap might be explained by the participation of both factors in the activation of the same genes, KDM2D by mediating H3K4 methylation and KDM6A by removing the repressive H3K27me mark. More striking, patients with characteristics of Kleefstra syndrome harbor alterations in *EHMT1* or *KMT2C* genes, involved in gene repression and gene activation, respectively. In a similar way, mutations in *NSD1* or *EZH2* cause overgrowth syndromes. This overlap in phenotype is in contrast with alterations in the different members of the MLL family of H3K4 methyltransferases (*KMT2A-D, SET1A*, and *SET1B*) that cause different symptoms, suggesting that they play crucial yet non-redundant roles in the brain. Finally, both gain and loss-of-function mutations in *NSD2* have been found in patients with intellectual disabilities [19].

Histone acetylation is involved in transcriptional activation, and it is controlled by the action of histone acetyltransferases (HATs) and histone deacetylases (HDACs). The acetylated lysine residues of histones are recognized by bromodomain (BRD)-containing proteins that function as effectors of the acetylation signal through the recruitment of factors that mediate transcription. Alterations in activities related to histone acetylation also cause neurodevelopmental disorders, including the loss of function of HATs, HDACs, BRD-containing proteins, and structural components of HAT complexes (Table 3) [16]. Similar to KMTs and despite the fact that multiple HATs seem to acetylate the same residues in histone tails, some non-overlapping symptoms have been described, suggesting that their functions are non-redundant. In addition, it is important to take into account that histone-modifying enzymes might also modify non-histone proteins, such as transcription factors that impact the epigenome.

In addition to histone modifications and its effector readers, gene expression and repression entail the remodeling of chromatin, making it more or less accessible to transcription factors and the transcriptional machinery. Chromatin remodelers utilize energy from ATP hydrolysis to alter nucleosome spacing/density or to facilitate histone variant exchange. Several activities with ATP-remodeling activity or that are components of ATP remodeling complexes have been identified in patients with rare diseases, the most well-known being the Coffin–Siris syndrome caused by loss-of-function mutations of different subunits of the SWI/SNF chromatin remodeling complex involved in transcriptional activation (Table 4).

Recently, it has been described that mutations in histone H3 tails can also contribute to rare neurologic dysfunctions and congenital anomalies. These mutations likely cause disruptions of H3 interactions with DNA, other histones, and histone chaperone proteins, and result in altered histone modification patterns [20].

## 3. Challenges in the Diagnosis of Rare Diseases

Patients affected by rare diseases can spend an average of 5 years looking for a diagnosis [4]. Initially, patients are tested for the most common genetic alterations that match their symptoms. If negative, patients often enter diagnostic programs that perform whole-exome (WES) and/or whole-genome (WGS) sequencing to identify genetic variants responsible for their disease. Despite the great improvements in diagnostics achieved by WES and WGS, these approaches still have many limitations.

WES can capture protein-coding regions of the genome, and in some cases, untranslated regions (UTRs) and intron-exon boundaries. It has lower costs than WGS and its analysis is more straightforward. However, WES covers only about 1–2% of the entire genome and has difficulties in detecting structural variants (variants that are greater than 50 base pairs and up to 3Mb), tandem repeats, and pathogenic variants in deep intronic regions and regulatory non-coding regions. Some of these challenges can be addressed by WGS; however, this technique also has its limitations, such as higher costs, similar limitations in structural variant detections, and more complex analyses.

Despite the great advances in diagnostic achieved by WES and WGS, more than 50% of patients might not receive definitive diagnoses after applying these technologies [21]. Complex rearrangements might remain undetected by short-read WGS. This limitation might be overcome by the use of long-read sequencing and novel optical genome mapping methods [22]. In addition to these limitations, many patients do not carry a variant previously reported to be associated with their symptoms. Instead, genome sequencing often reveals a large number of candidate variants whose implications in diseases are unknown and, therefore, are called variants of unknown significance (VUS). Compared to exonic variants, the interpretation of noncoding variants is far more challenging. The transcriptomic and epigenomic approaches discussed here might help to interpret these variants.

## 4. Epigenetic and Functional Approaches for Rare Diseases Diagnosis

Genetic studies often reveal a large number of VUS. More recently, functional approaches to identify or confirm variants involved in diseases have been developed. Some of them are focused on correlating variants with alterations in gene expression or epigenomic marks.

### 4.1. Choice of Cells and Tissues

Gene expression and its regulatory mechanisms, different from genetics, vary from tissue to tissue; therefore, the choice of tissue or cell type to carry out functional approaches is critical. However, certain tissues might be difficult to access or might be unrealistic for undiagnosed disease programs covering hundreds of patients with different phenotypes. The most common tissues collected from patients for diagnostic purposes are blood followed by skin. Although most studies analyze whole blood, purifying mononucleated cells or other subpopulations of cells might bring some advantages. When analyzing whole blood, it is important to take into account that expression and epigenetic patterns are cell-specific; therefore, the differences found in patients might reflect changes in cellular composition. Blood offers the possibility to generate patient-derived B-lymphoblastoid cell lines (LCLs), which consist of transforming B lymphocytes with the Epstein–Barr virus (EBV). LCLs are immortalized cell lines and can be used for follow-up studies. Skin offers the opportunity to stablish primary cultures of fibroblast or keratinocytes. In any case, long passages of patient-derived cell lines should be avoided to minimize the chance of introducing genetic aberrations.

Other tissues that are relatively easy to access are skeletal muscle or fat, but others are more difficult to obtain and might be only available if a therapeutic surgery is performed. One way to preserve access to such precious material is to establish organoid cultures, self-organized three-dimensional tissue cultures that replicate much of the complexity of an organ and that can be indefinitely expanded. Importantly, protocols for the establishment of organoids from a large variety of human tissues have been reported [23]. Changing the identity of an available cell type to another is an additional strategy that can be used to obtain hardly accessible tissues or cell types. Multiple cell types, including neurons, adipocytes, myocytes, and pancreatic cells can be obtained by overexpressing certain transcription factors in human fibroblasts [24]. The patient’s somatic cells can also be reprogrammed to pluripotency by overexpressing transcription factors. These induced pluripotent cells (iPSCs) can be differentiated in vitro to virtually any cell type [24].

### 4.2. Transcriptomic Profiles by RNA-seq

Transcriptomics profiles have been successfully used in the diagnosis of rare diseases. The sequencing of transcripts (RNA-seq) allows the identification of aberrantly expressed genes, aberrant splicing, monoallelic expression, and variant identification, including structural variants. Therefore, RNA-seq can improve the interpretation of VUS identified by genotyping.

Regarding aberrantly expressed genes, RNA-seq has been useful for the identification of underexpressed genes most commonly affected by frameshifts, truncations, and splicing mutations that induce mRNA nonsense-mediated decay. Missense mutations appear less likely to be correlated with altered mRNA levels. Additionally, unexpected increases in mRNA levels have been reported for some mutant genes not producing proteins and might reflect a compensatory mechanism in response to absent protein [8]. RNA-seq has also been successfully used for variant calling [19]. This approach limits the detection of variants to genes that are expressed in the analyzed tissue and, therefore, is not intended to replace WGS or WES approaches but rather offer an alternative in cases where they are neither available nor cost-effective. In addition, RNA-seq can be used to identify VUS that affect splicing, especially those that introduce synonymous mutations at exons and variants at introns that might have not been prioritized in the genomic analysis. The allele-biased expression can also be identified by RNA-seq, pointing to the presence of structural variants, single nucleotide variations (SNVs), or imprinting defects that alter the expression of one allele.

Therefore, compared to WES or WGS, RNA-seq can provide a functional assessment of genetic variation but it also implies additional challenges that will be discussed next.

#### 4.2.1. Tissue-Specific Expression

An essential limitation of RNA-seq approaches is the fact that each tissue expresses only a subset of genes. Analysis of the expression of disease-associated genes in different human tissues has shown that mitochondrial disease genes are the most ubiquitously expressed, but other disease-associated genes have more pronounced tissue-specific expression profiles, such as neurological genes in the brain [25]. Analysis of gene expression across 49 tissues and cell types showed that fibroblasts were the cell types expressing the highest number of Mendelian disease genes while muscle tissue expressed the lowest, except for neuromuscular disorder-associated genes [25]. Although obtaining a skin biopsy is more invasive than blood extraction, skin-derived fibroblasts appear to be a more useful resource, showing a higher number of expressed genes and less variability between samples than blood, likely explained by the heterogeneity of cell types found in blood [25,26]. In addition, fibroblast expression patterns are more similar to muscle than blood and would be preferred for the diagnosis of neuromuscular diseases [27]. However, blood-derived LCLs have been described as doubling the number of genes expressed in blood and have been successfully used to identify aberrant splicing events in undiagnosed patients that matched the Cornelia de Lange phenotype [28]. Transdifferentiation strategies have also been used to solve the unavailability of biopsies. Fibroblasts from patients with muscular disorders were transdifferentiated to myotubes by MyoD overexpression. These engineered myotubes shared a significant expression profile with the skeletal muscle and allowed the detection of splicing aberrations in genes involved in muscular diseases that were not expressed in blood or fibroblasts [27]. Other strategies have been oriented to improve the read depth of poorly expressed transcripts by depleting highly expressed transcripts, such as hemoglobin transcripts in the blood or the use of Cas9 to remove unwanted high-abundance species in sequencing libraries [29,30].

#### 4.2.2. Source of Control Healthy Samples

An additional challenge of transcriptomic approaches for diagnosis is the need to compare patients’ samples with healthy controls. In some scenarios, the inclusion of a reasonable number of healthy samples is possible. For example, Hong et al. compared patients’ muscle biopsies with muscle control samples obtained from healthy individuals undergoing plastic surgery [31]. However, in most cases, healthy tissues are even more problematic to obtain than patients’ tissues. One potential solution is to use RNA-seq data published by others as healthy controls. A great source of mRNA expression data in different human tissues is provided by the Genotype–Tissue Expression sequencing project (GTEx) (https://www.gtexportal.org/ accessed on 25 June 2022) [32]. Interestingly, the GTEX portal allows the selection of samples that better match the query cohort regarding age or sex. However, disparities in library preparation and sequencing strategies typically introduce variability that might compromise the identification of alterations in patients, especially when assessing differential expression. Although normalization strategies focused on overcoming the variability across sequencing batches have been developed [33,34,35,36], sources of variability should be avoided as much as possible. For example, Cumming et al. reduced variability between query and control samples by sequencing patients’ samples using the same protocol as the GTEx project and analyzing the data using identical pipelines to minimize technical differences. In this way, they identified rare splicing events present in muscle samples of patients with rare muscle disorders but not present in the GTEX of healthy muscle samples [37]. A similar approach was used to identify splice junctions and rare variants in LCL cell lines from patients with Cornelia de Lange symptoms that were not present in healthy LCLs and blood samples from the GTEX collection [28]. An alternative successful strategy used to overcome the lack of appropriate control samples when using large cohorts of patients consists of comparing one patient against the rest of the patients that would serve as controls [26,38].

#### 4.2.3. Expression of Outliers versus Global Expression Changes

Studies that have focused on the identification of expression outliers have typically pinpointed two to three outliers per patient with significantly increased or decreased expression [26]. However, patients with a substantial number of differentially expressed genes have also been identified [25]. Abnormal expressions of several genes adjacent to each other are suggestive of possible contiguous deletion [11]. More commonly, the disease causative gene might produce downstream effects that can be reflected in the transcriptome (Figure 1), providing functional evidence that can guide or support diagnostic interpretation. A recent report shows that the loss of function of about 30% of tested genes in the cancer cell line K562 results in a transcriptional phenotype with more than 10 differentially expressed genes (DEGs) [39]. In accordance, Yépez et al. found a lower abundance of mitochondrial transcripts while analyzing the fibroblasts transcriptome of a patient with suspected mitochondrial disease. This finding confirmed the involvement of mutations in *LRPPRC*, a gene that regulates the stability of mature mitochondrial transcripts, in the patient phenotype [25]. Similarly, in another patient, a high number of downregulated mtDNA genes supported a functional defect of *LIG3*, a gene causing mtDNA depletion when mutated. Therefore, the analysis of pathways and function enrichment in DEGs can be helpful to support diagnostics. This might be particularly relevant when mutations affect transcription factors, chromatin-related factors, or activities involved in signal transduction that impinge on transcriptional pathways (Figure 1). In these situations, it is expected that a substantial number of genes change expression. In this regard, the description of detailed transcriptomic signatures associated with a disease in common tissues, such as blood or fibroblasts, might be useful for the diagnosis of future patients. In addition, Hong et al. used RNA-seq data to perform clustering of patients with undiagnosed neuromuscular diseases based on their gene expression data to identify patients with similar pathologies [31]. The analysis of the pathways enriched in each cluster helped to identify common altered functions. Moreover, the enrichment in certain pathways, such as metabolic, inflammatory, or stress response pathways might not only provide a clue about the patient’s pathology but also the opportunity to target a biological pathway for treatment.

#### 4.2.4. Single Cell Transcriptomics

An additional source of sample variability that might be explored to facilitate diagnosis is the heterogeneity in the cellular composition of biopsies. Single-cell transcriptomics (scRNA-seq) can allow the detection of transcripts expressed in rare populations of cells, evaluate the abundance of different cell types, or tackle mosaicism in a biopsy. However, its implementation for diagnostic purposes is not feasible at present regarding cost and analysis efforts. Related approaches have aimed to extrapolate cellular components from bulk RNA-seq using deconvolution methods. Hong et al., applying these methods, deconvoluted cell type abundances in muscle biopsies from patients with neuromuscular diseases and captured clinical and pathological aspects of the diseases. For example, the abundance of fibro–adipogenic progenitor cells estimated from the deconvolution of the bulk RNA-seq data correlated with muscle fibrosis in patients [31].

#### 4.2.5. Success Rate

Overall, RNA-seq used to identify expression and/or splicing outliers has been reported to provide diagnoses for about 10–20% of undiagnosed cases with negative WES or WGS and confirmed diagnosis in around 50% of cases with a candidate variant identified by genome sequencing [26,37,38]. The highest diagnosis ratios are achieved when focusing on one particular pathology and analyzing the corresponding affected tissue, such as muscular disorders and muscle biopsies [31]. Overall, detection of splicing aberrations appears more successful than identifying causative genes by differential expression. This might be due to the fact that many mutations do not alter the mRNA levels of genes but might also reflect the difficulties in identifying differentially expressed genes when using different batches of sample preparation. The most successful reported scenario appears to involve cases of compound heterozygosity in which one pathogenic variant is identified by WES and the second variant is confirmed by aberrant splicing detected in the RNA-seq. Despite not offering a diagnosis right away, in many cases, the transcriptomic analysis provides several promising expression- and splicing outlier candidate genes in which a complete genetic diagnosis is yet to be confirmed.

### 4.3. DNA Methylation

A number of publications have described changes in DNA methylation profiles associated with genetic syndromes. This has allowed the development of strategies that allow the classification of undiagnosed patients in one particular disease according to their methylation profile in blood. Simple and cost-effective genome-wide DNA methylation arrays, such as the Illumina Infinium HumanMethylation450 or HumanMethylationEPIC BeadChip array, assess the methylation status of approximately 450,000 and 850,000 CpGs.

Pipelines that allow the classification of patients into a number of syndromes according to their DNA methylation profiles have been developed. The EpiSign classifier is based on 100–500 differentially methylated probes that best separate the case samples from controls and allow the diagnosis of undiagnosed cases based on those episignatures [40]. The patients’ methylation profiles are contrasted with a clinical database with thousands of peripheral blood DNA methylation profiles, including disorder-specific reference cohorts and normal samples. EpiSign currently screens for a total of 74 neurodevelopmental syndromes, including 7 imprinting disorders and 2 trinucleotide repeat expansion disorders [40,41]. These signatures are not only associated with mutations in genes involved in DNA methylation but also with other genes, such as histones and genes involved in histone modifications, chromatin remodeling, splicing, copy-number variation, cohesin-related functions, mitochondrial functions, ubiquitin-conjugating enzymes, transcription factors, and copy number variation [40]. Moreover, for certain syndromes, the episignature can be nailed down to mutations in a particular domain of a gene.

In addition, public resources for the analysis of DNA methylation data, such as the EpigenCentral portal (https://epigen.ccm.sickkids.ca/ accessed on 25 June 2022), have been recently developed [42]. This free web resource allows the classification of patients with rare diseases into 10 neurodevelopmental syndromes by uploading their blood methylation patterns obtained using the HumanMethylation450 or HumanMethylationEPIC BeadChip. In addition, it allows the identification of differentially methylated regions (DMR) between submitted samples.

Despite the reported success of using DNA methylation patterns for the diagnosis of complex cases, this approach has several limitations. First, the study is focused on blood samples from neurodevelopmental cases and, therefore, is expected to be limited to patients with germline mutations in genes that are expressed in blood and that have an impact on its methylome. Neurodevelopmental syndromes caused by alterations in neuronal-specific genes, such as neurotransmitters carriers and transporters, are not expected to confer a particular methylation pattern in blood. Another limitation is the need to develop unique analytical methylation profiles for each Mendelian disorder, requiring expansion of reference databases and the development of sophisticated, machine-learning-based bioinformatic algorithms [40]. Sources of variation, such as underrepresented ethnicities, also need to be taken into account. Similar to transcriptomic analysis, the analysis of blood DNA methylation is conducted in bulk, and it is expected that syndromes that alter the blood cellular content might also reflect changes in DNA methylation.

In addition to genome-wide changes in DNA methylation, pathogenic genetic alterations might disrupt DNA methylation at one particular site of the genome. Barbosa et al. found that 20% of patients with neurodevelopmental diseases of unknown causes carried one rare epigenetic change specific to one allele [43]. A few of these epivariations were found at the promoters of genes known to show altered methylation in congenital diseases. Additionally, they found hypermethylation that correlated with expansions of GC-rich tandem repeats and loss of methylation in imprinted loci. Copy number and single nucleotide variations were found in the vicinity of these epivariations, suggesting that they might occur secondarily to an underlying regulatory sequence mutation. Interestingly, some of these SNVs disrupt CCCTC-binding factor (CTCF) binding motifs, transcription factors with roles in chromatin organization. In agreement with this finding, a more recent study identified SNVs that disrupted TFBSs associated with outlier DNA methylation profiles and altered the expression of nearby genes in individuals with congenital heart defects [44]. However, it is important to take into account that differentially methylated regions not associated with genetic variation (and that were likely sporadic) were also identified.

Similar to DNA methylation, mutations in histone modifiers and mutations in histone tails are expected to alter the patterns of histone modifications. Global patterns of histone modifications are characterized by chromatin immunoprecipitations coupled to sequencing (ChIP-seq). Compared to DNA methylation arrays, ChIP-seq is a far more tedious and variable technique, which so far has not been implemented for rare disease diagnosis routines.

### 4.4. Detection of Regulatory Variants

Studies investigating the genetic basis of rare diseases (focused on coding variants) have failed to provide clear answers for more than 40% of the studied cases [21], suggesting that a large proportion of cases may be caused by alterations outside of the coding regions. Among other effects, non-coding genetic alterations can have dramatic effects on the expression of genes by altering the functionality of enhancer regions.

Enhancers are distal regulatory units that participate in the regulation of gene expression by establishing contacts with promoters favoring the recruitment of RNA polymerase II. Enhancers contain docking sites for transcription factors (TFs) that in many cases are tissue-specific. These transcription factors participate in the recruitment of HATs and KMTs that maintain high levels of histone acetylation and monomethylation of lysine 4 of histone H3 (H3K4me1) (Figure 1). The abundance of transcription factor binding activity in enhancers promotes a relaxed and accessible chromatin configuration that can be detected using chromatin accessibility techniques, such as DNA-seq or ATAC-seq [45].

Enhancer dysfunction might be caused by alterations in chromatin-related factors causing global deregulation of chromatin and gene expression. Alternatively, patients might carry mutations that genetically disrupt the enhancer region, including SNVs that alter TFBSs, and that affect, in this way, histone modifications and/or DNA methylation (Figure 1). Genetic alterations in enhancer regions have been identified in several diseases. A rare case of aniridia was nailed down to a de novo point mutation in an enhancer located 150 kb downstream from *PAX6* that disrupts an autoregulatory PAX6 binding site [26]. Structural variants on a gonad-specific *SOX9* transcriptional enhancer caused the aberrant gonadal expression of SOX9, causing a disorder of sex development [27]. Recessive mutations in a developmental enhancer of *PTF1A* were found in patients with isolated pancreatic agenesis [28]. A homozygous point mutation in a highly conserved enhancer region downstream of the developmental transcription factor *TBX5* has been reported in patients with congenital heart disease [46]. Moreover, point mutations were found on an enhancer controlling the expression of the gene *SHH* causing preaxial polydactyly [47].

Most of the reported regulatory alterations mentioned above were identified by focusing on the regulatory regions of well-known disease-causing genes in patients with very specific pathologies. Unfortunately, identifying disease-causing non-coding variants at enhancer regions in large cohorts of undiagnosed patients with variable symptoms might be challenging. Eventually, larger variants may be more disruptive to regulatory elements than SNVs and, therefore, easier to predict their pathogeny. Turró et al. focused on identifying large deletions likely to disrupt enhancer functions in a cohort of patients with hematopoiesis-related disorders [48]. First, they identified active regulatory elements in six hematological cell types by merging transcription factor binding sites with chromatin accessibility data and regions marked with histone acetylation. These regulatory elements were mapped to disease-relevant genes using chromosome conformation capture coupled with sequencing (Hi-C) to identify enhancer–promoter interactions. In three cases, large deletions that overlapped with the identified disease-relevant enhancers correlated with altered gene expression that explained the patients’ phenotypes.

It has been estimated that 1–3% of neurodevelopmental patients without a diagnostic coding variant carry pathogenic de novo mutations in fetal brain-active enhancers [32]. However, despite some successful reports, inferring how genetic variations can affect enhancer functions, gene expression, and disease is currently challenging. First, the identification of enhancer regions is not straightforward. Although the most common criteria to identify these regions are based on the presence of certain chromatin marks, high chromatin accessibility, and concentration of TFBSs, there are no unifying criteria to identify enhancers at present [49]. Second, identifying the genes regulated by one particular enhancer is also challenging. Target genes might be located far away, and one enhancer might regulate several genes, giving rise to complex phenotypes. Recently, the refinement of chromosome conformation capture technologies, such as Hi-C, has significantly improved the detection of promoter–enhancer interactions. In addition, genome-wide association studies (GWAS) using large populations have identified expression quantitative trait loci (eQTL) that correlate with the expression of genes and rare variants associated with gene expression outliers [50]. Third, compared to coding or splicing mutations, enhancer alterations can affect the expression of genes in a tissue-specific manner. In the most challenging scenario, the effects can be developmental-stage specific and may not be detected in adult-differentiated tissue. Moreover, for enhancers active only in certain populations of cells in bulk, an analysis of biopsies might challenge their identification. To solve some of these problems, human embryonic stem cells (hESC) or iPSCs can be used to generate disease-relevant cell types that are otherwise difficult to obtain. For example, enhancer epigenomic annotation in hESC-derived pancreatic progenitor cells has been used to guide the interpretation of whole-genome sequences from individuals with isolated pancreatic agenesis [51]. Finally, strategies to validate predicted enhancers are tedious and time-consuming, although the recent introduction of CRISPR/Cas9 strategies has opened up new opportunities to confirm enhancer activity and investigate non-coding variants located in cis-regulatory elements [52].

Genetic variation can also influence the 3D organization of the genome in the nucleus, resulting in the dysregulation of gene expression and, consequently, might cause disease. Groups of adjacent coregulated genes, often targeted by common enhancers, have been described to cluster within megabase-scale topological associating domains (TADs) [53]. TADs are separated by boundary regions that act as insulators that block interactions across different TADs. Structural variations, such as deletions, inversions, or duplications have the potential to interfere with the TAD structure by disrupting or repositioning its boundaries. Disruption of TAD boundaries can lead enhancers to interact with genes outside of the TAD, which can contribute to congenital disorders, including limb malformation [54]. TAD disruption might also explain conditions caused by balanced translocations or rearrangements without gene alterations in which deletion or misplacement of TAD boundaries allow enhancers from neighboring domains to ectopically activate genes. In addition, TADs boundary regions often contain CTCF binding sites, whose disruptions are predicted to alter TAD interactions. As described before, CTCF binding sites have been reported to affect DNA methylation when disrupted by rare SNVs, suggesting that alterations of TADs may also impact DNA methylation [43].

## 5. Conclusions and Perspectives

Here, we discussed epigenetic and functional strategies for the diagnosis of complex cases of rare diseases. It is becoming clear that the implementation of multiple strategies is typically needed to reach a diagnosis in complex cases. In this sense, epigenetic strategies are intended to complement genomic techniques, such as WES and/or WGS. These techniques can be used to confirm the pathogenicity of a VUS already identified after genome sequencing or identify variants that are not prioritized after the genomic analysis. However, several aspects need to be improved before they can reach their full diagnosis potential. A better description of altered patterns of DNA methylation, histone modifications, and gene expression changes for each disease is needed to improve the classification of patients into one particular syndrome. Still, the functions of many human genes are unknown and the transcriptional or epigenetic phenotypes resulting from their perturbation remain undescribed. The interpretation of VUS in non-coding regions is more challenging. In this regard, a better description of the regulatory regions that control the expressions of disease-relevant genes in each tissue or cell type will be fundamental to anticipate the consequences of their malfunctions. In addition, there is a need for improved sequencing methods with better coverage and accuracy, but also with lower costs and more accessibility. Making the best of sequencing patients’ data requires improved machine learning techniques for more successful classifications of patients. However, the success of these approaches might be limited by the small number of patients affected by each pathology. Data sharing and collaborative efforts should be oriented to overcome this limitation. Novel methods, such as scRNA-seq, can improve our understanding of rare diseases but are far from being used as routine diagnosis methods due to cost and analysis challenges. Overall, there is a need for realistic approaches that can be implemented by diagnostic programs around the world that deal with multiple complex cases with variable pathologies. For that, affordable, sustainable, and accessible standardized methods need to be developed to ensure equal access to diagnosis for all patients.

## Figures and Tables

**Table 1:** DNA methylation-related genes known to cause rare diseases according to OMIM (https://www.omim.org/ accessed on 25 June 2022).

Function	Gene Symbol	Disease	MIM Phenotype
DNMT	*DNMT1*	Cerebellar ataxia, deafness, narcolepsy, autosomal dominant	604121
		Neuropathy, hereditary sensory, type IE	614116
	*DNMT3A*	Heyn–Sproul–Jackson syndrome	618724
		Tatton–Brown–Rahman syndrome	615879
	*DNMT3B*	Facioscapulohumeral muscular dystrophy 4, digenic	619478
		Immunodeficiency–centromeric instability–facial anomalies syndrome 1	242860
MBD- containing protein	*MECP2*	Rett syndrome	312750
	*MBD5*	Intellectual developmental disorder, autosomal dominant 1	156200
	*GATAD2B*	GAND syndrome	615074

**Table 2:** Genes involved in histone methylation known to cause rare diseases according to OMIM (https://www.omim.org/ accessed on 25 June 2022).

Function	Gene Symbol	Disease	MIM Phenotype
H3K4 KMT	*KMT2A*	Wiedemann–Steiner syndrome	605130
	*KMT2D*	Kabuki syndrome type 1	147920
	*KMT2C*	Kleefstra syndrome 2	617768
	*KMT2B*	Dystonia 28, childhood-onset	617284
	*SET1A*	Epilepsy, early-onset, with or without developmental delay	618832
		Neurodevelopmental disorder with speech impairment and dysmorphic facies	619056
	*SET1B*	Intellectual developmental disorder with seizures and language delay	619000
	*ASH1L*	Intellectual developmental disorder, autosomal dominant 52	617796
H3K9 KMT	*EHMT1*	Kleefstra syndrome 1	610253
H3K27 KMT	*EZH2*	Weaver syndrome	277590
H3K36 KMT	*NSD1*	Sotos syndrome	117550
	*NSD2*	Rauch–Steindl syndrome	619695
	*SETD2*	Luscan–Lumish	616831
H4K20 KMT	*KMT5B*	Intellectual developmental disorder, autosomal dominant 51	617788
H3K4 KDM	*KDM1A*	Cleft palate, psychomotor retardation, and distinctive facial features	616728
	*KDM5C*	Intellectual developmental disorder, X-linked syndromic, Claes–Jensen type	300534
H3K27 KDM	*KDM6A*	Kabuki syndrome type 2	300867
H3K9 KDM	*PHF8*	Intellectual developmental disorder, X-linked, syndromic, Siderius type	300263

**Table 3:** Genes involved in histone acetylation known to cause rare diseases according to OMIM (https://www.omim.org/ accessed on 25 June 2022).

Function	Gene Symbol	Disease	MIM Phenotype
HATs	*KAT6A*	Arboleda–Tham syndrome	616268
	*KAT6B*	Genitopatellar syndrome	606170
		SBBYSS syndrome	603736
	*CREBBP*/ *EP300*	Rubinstein–Taybi syndrome	180849
		Menke–Hennekam syndrome 2	618333
BRD-containing protein	*BRPF1*	Intellectual developmental disorder with dysmorphic facies and ptosis	617333
HDAC	*HDAC4*	Neurodevelopmental disorder with central hypotonia and dysmorphic facies	619797
	*HDAC8*	Cornelia de Lange syndrome 5	300882
BRAF complex subunit	*PHF21A*	Intellectual developmental disorder with behavioral abnormalities and craniofacial dysmorphism with or without seizures	618725
HAT complex subunit	*TRRAP*	Developmental delay with or without dysmorphic facies and autism	618454

**Table 4:** Genes involved in chromatin remodeling known to cause rare diseases according to OMIM (https://www.omim.org/ accessed on 25 June 2022).

Function	Gene Symbol	Disease	MIM Phenotype
SWI/SNF complex	*ARID1A*	Coffin–Siris syndrome 2	614607
	*ARID1B*	Coffin–Siris syndrome 1	135900
	*ARID2*	Coffin–Siris syndrome 6	617808
	*SMARCB1*	Coffin–Siris syndrome 3	614608
	*SMARCA4*	Coffin–Siris syndrome 4	614609
	*SMARCE1*	Coffin–Siris syndrome 5	616938
	*ARID2*	Coffin–Siris syndrome 6	617808
	*DPF2*	Coffin–Siris syndrome 7	618027
	*SMARCC2*	Coffin–Siris syndrome 8	618362
	*SMARCD1*	Coffin–Siris syndrome 11	618779
	*SMARCD2*	Specific granule deficiency 2	617475
	*ATRX*	Alpha-thalassemia/mental retardation syndrome	301040
		Intellectual disability-hypotonic facies syndrome, X-linked	309580
ISWI complex	BPTF	Neurodevelopmental disorder with dysmorphic facies and distal limb anomalies	617755
CHD family	*CHD2*	Developmental and epileptic encephalopathy 94	615369
	*CHD7*	CHARGE syndrome	214800
		Hypogonadotropic hypogonadism 5 with or without anosmia	612370
	*CHD8*	Intellectual developmental disorder with autism and macrocephaly	615032
	*CHD5*	Parenti–Mignot neurodevelopmental syndrome	610771
	*CHD1*	Pilarowski–Bjornsson syndrome	617682
	*CHD3*	Snijders Blok–Campeau syndrome	618205
	*CHD4*	Sifrim–Hitz–Weiss syndrome	617159
